# Fully automated rodent brain MR image processing pipeline on a Midas server: from acquired images to region-based statistics

**DOI:** 10.3389/fninf.2013.00015

**Published:** 2013-08-13

**Authors:** Francois Budin, Marion Hoogstoel, Patrick Reynolds, Michael Grauer, Shonagh K. O'Leary-Moore, Ipek Oguz

**Affiliations:** ^1^Neuro Image Research and Analysis Laboratories, Department of Psychiatry, University of North CarolinaChapel Hill, NC, USA; ^2^Kitware, Inc.Carrboro, NC, USA; ^3^Fetal Toxicology Laboratory, Department of Psychiatry, Bowles Center for Alcohol Studies, University of North CarolinaChapel Hill, NC, USA

**Keywords:** rodent, magnetic resonance imaging, automatic processing, server

## Abstract

Magnetic resonance imaging (MRI) of rodent brains enables study of the development and the integrity of the brain under certain conditions (alcohol, drugs etc.). However, these images are difficult to analyze for biomedical researchers with limited image processing experience. In this paper we present an image processing pipeline running on a Midas server, a web-based data storage system. It is composed of the following steps: rigid registration, skull-stripping, average computation, average parcellation, parcellation propagation to individual subjects, and computation of region-based statistics on each image. The pipeline is easy to configure and requires very little image processing knowledge. We present results obtained by processing a data set using this pipeline and demonstrate how this pipeline can be used to find differences between populations.

## Introduction

Magnetic Resonance (MR) Imaging studies are among the increasingly popular methods of assessing neurodevelopment in rodents. MRI is a non-invasive method used to study the brain's anatomical structure and connectivity without any known biological effect on the tissues. It is a highly translational technique because it is used both in clinical settings as well as in pre-clinical animal research and data can be acquired using similar methods in both humans and animals. The use of rodent models for translational animal studies of clinical disorders has allowed researchers to study precise effects of drug treatments or exposure to specific substances in a defined environment for all subjects, while minimizing uncontrolled external factors. There is also an increasing interest in the scientific community for the use of MRI to enable large-scale genetic phenotyping studies.

A typical image analysis study consists of comparing the differences between an experimental group and a control sample. One common method is to perform a region-based analysis of the MR Images. The average and standard deviation of properties, such as the volume or the intensity of each region, is used to compare the different populations (Harms et al., 2006; Stone et al., 2008; Wang et al., 2008; Chan et al., 2009; Fatemi et al., 2009; Lodygensky et al., 2009; Zahr et al., 2009; Badea et al., 2010; Hui et al., 2010). Structural Magnetic Resonance Imaging (sMRI) and Diffusion Tensor MRI (DTI) can be used in such studies. While sMR images are relatively straightforward to analyze, DTI data require additional processing. The latter are estimated from Diffusion Weighted Imaging (DWI) scans, which map the diffusion process of the water molecules in biological tissues. Each voxel of the image contains a 3 × 3 symmetric definite positive matrix that can be visualized as an ellipsoid. A number of scalar measurements can be computed from the DTI data, including Fractional Anisotropy (FA), which represents how directionally restricted along one axis the diffusion is (between 0, no directionality, and 1, diffusion in only one direction), Mean Diffusivity (MD), which quantifies the total diffusion within a voxel, Radial Diffusivity (RD), a measure of the average diffusion along the 2nd and 3rd diffusion axis, and Axial Diffusivity (AD), diffusion along the main direction. It has been shown that these measurements are valuable clinical tools that can be used to diagnose tissue deficiencies or delineate biological differences (van Gelderen et al., 1994; Pierpaoli and Basser, 1996; Nair et al., 2005; Song et al., 2005; Sun et al., 2005; Zhang et al., 2006). In addition to the scalar measurements, images acquired without a diffusion gradient, i.e., the b = 0 images, and the average of all the images acquired with a non-zero gradient, i.e., the Isotropic Diffusion Weighted Image (iDWI), which are extracted from the DWI scans, can also be useful to detect brain lesions (Moon et al., 2007).

Analyzing data from this type of study typically necessitates a number of steps: a registration step to align all images in the same space, a skull-stripping step to eliminate all non-brain tissues that are not being studied and several other steps which may include segmentation of the brain into different Regions Of Interest (ROI), also called parcellation, so that their properties (e.g., average intensity and volume) can be analyzed. Although each of these steps can be performed manually for each image, this is labor intensive and subject to inter- and intra-rater variability, which can often confound potentially subtle group differences in the data set.

Due to the need for these sophisticated analyses, DTI processing is inaccessible to small animal researchers with limited image processing experience. For this reason, it is imperative to build user-friendly software that implements automated pipelines to process the data.

A number of automatic pipelines and set of tools to perform those aforementioned steps are available for human data, such as FSL^1^ (Smith et al., 2004), the LONI pipeline^2^ (Dinov et al., 2010) which integrates tools such as BrainSuite^3^ (Shattuck and Leahy, 2002) and which could be used to create a custom pipeline, and FreeSurfer^4^ (Fischl et al., 2002), but these produce very limited results when applied to rodent scans. Registration parameters in these tools are not appropriate due to image scale and ratio differences. Skull-stripping algorithms produce poor results due to non-appropriate assumptions regarding the data (shape of the skull, space between brain and skull, etc).

Numerous individual tools, such as AtlasWerks^5^, which creates an average image from a set of images using diffeomorphic registration, and ANTs^6^, a software package that performs image to image diffeomorphic registration, are also available but each of them performs only a small part of the whole analysis process. Moreover, most of the default parameters of those tools are set for the human brain, thus not useful for direct application for the rodent brain.

The image processing method we are presenting in the paper has been used by different groups conducting research on rodent images and has been described in previous publications (Chen et al., 2005; Kovacevic et al., 2005; Badea et al., 2007; Calabrese et al., 2013). Brain masks can be outlined manually (Lau et al., 2008) or computed automatically (Badea et al., 2007). The creation method for an average image has already been presented (Chen et al., 2005; Kovacevic et al., 2005; Badea et al., 2007; Lau et al., 2008; Lerch et al., 2008; Calabrese et al., 2013). This step is performed using existing tools such as ANTs or the mni_autoreg^7^ tools (Collins et al., 1994, 1995). The parcellation of the average population image, that is manually or automatically done, is then propagated back to each subject using the inverse transform that was computed to create the average image (Lerch et al., 2008; Calabrese et al., 2013). Each tool is often run individually which makes the process cumbersome. Pipelines automatically computing all the steps described above have also been created (Badea et al., 2012). Although the existing processing steps are well known, well described and reproducible, they are by no means accessible to the typical preclinical researcher. First, most, if not all, the tools require command line processing, which is hardly user-friendly. Additionally, and perhaps more importantly, each tool comes with numerous parameters, whose meanings and interpretations require expertise in image processing algorithms. Setting the values of all these dozens of parameters is a task that is inhibitive to the typical preclinical researcher.

Our approach addresses these problems in three critical ways. First, our entire pipeline is accessible through a web browser, which is a familiar environment to even the most computer-illiterate researcher. Second, we provide carefully fine-tuned default settings to all the parameters, allowing the inexperienced user to use the functionality offered out-of-the-box. However, it should be noted that all the advanced parameters are also accessible if desired, to allow the more experienced user flexibility. Third, the Midas environment inherently allows data and processing sharing: this can be crucial for those occasions when the out-of-the-box does not work, and the user can ask for help from an image processing expert for troubleshooting. All this person would then need is to have access to the Internet, and they can see the processing steps that were applied, the input and output, and help pinpoint the problem. Compare this to the more traditional scenario of having to transfer dozens of images to this expert, and the non-expert trying to remember the exact details and sequence of processing that was already tried, the expert working on this on his/her own computer, then transferring the results back to the user, perhaps only to discover that they don't have access to the same version of the software, and having to start over.

In this paper we present a processing pipeline targeted toward a relatively inexperienced preclinical researcher who, for example, doesn't wish to learn about the intricate details of image registration algorithms but just wants to compute the volume of the caudate nucleus in his data. We propose to make this possible via our Midas-based rodent brain image processing pipeline. The user does not have to go through complicated steps to find all the individual tools necessary for the processing as well as parameters giving correct results for rodent images. We illustrate the usage of the different steps of our method (rigid registration, skull-stripping, average computation, average parcellation, individual parcellation and computation of region-based statistics) by processing a very simple dataset and present the results as well as the possibilities and the limitations of the developed pipeline.

## Methods

### Overview of the pipeline

**Figure 1:**
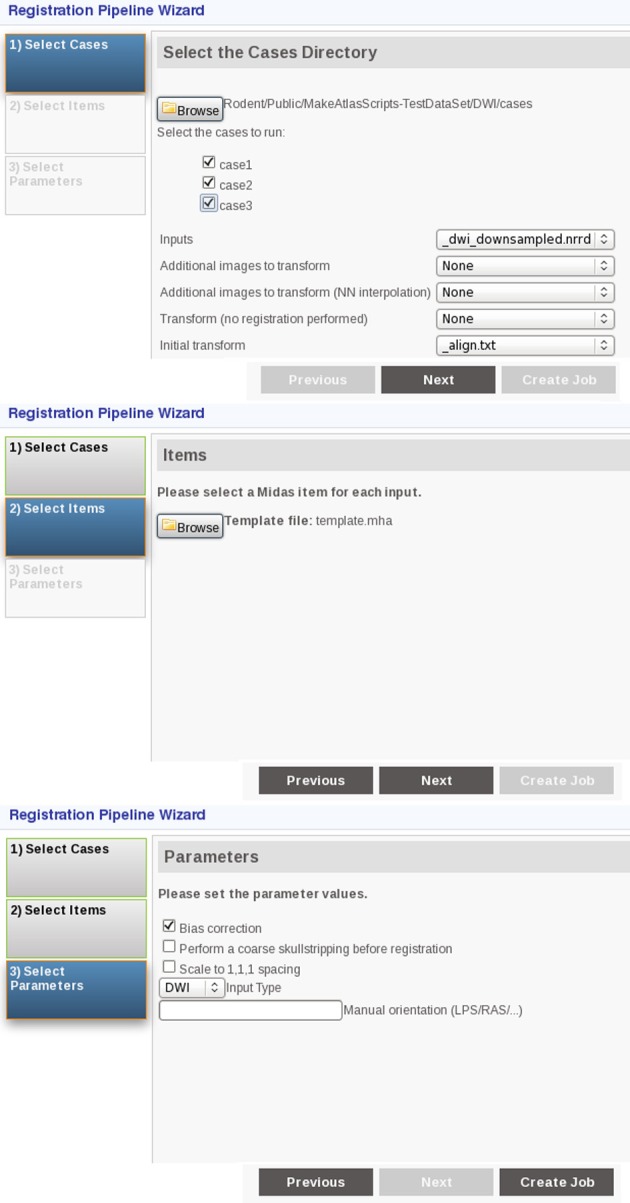
**Screenshots presenting the user interface of the pipeline when running the registration step**. From top to bottom: selection of the images to register, selection of the fixed image for the registration, selection of the options including the type of the input image.

The current pipeline was developed to perform a region-based analysis of 3D medical images and has been optimized for MR rodent brain imagery. It can process DWI, DTI and structural MRI data and is composed of a set of BatchMake^8^ scripts designed for a Midas^9^ server. Midas is a toolkit that enables web-enabled data storage and has been designed for large data such as medical images. It provides a mechanism to integrate plugins to perform actions such as processing and analyzing the images stored on the server. Such plugins can integrate BatchMake, a cross-platform scripting language and grid computing abstraction layer for processing data locally or on distributed system, thus providing computational power if installed on a computer grid. The Midas platform was chosen because it allows easy sharing of data among collaborators. The pipeline incorporates existing tools as well as some specific tools that were created to assemble all of the existing ones in a pipeline. To run it, the researcher uses the web interface to select the plugin to run, to choose the files to process, and to set the options (Figure 1). The final results are directly uploaded back onto the server.

**Figure 2:**
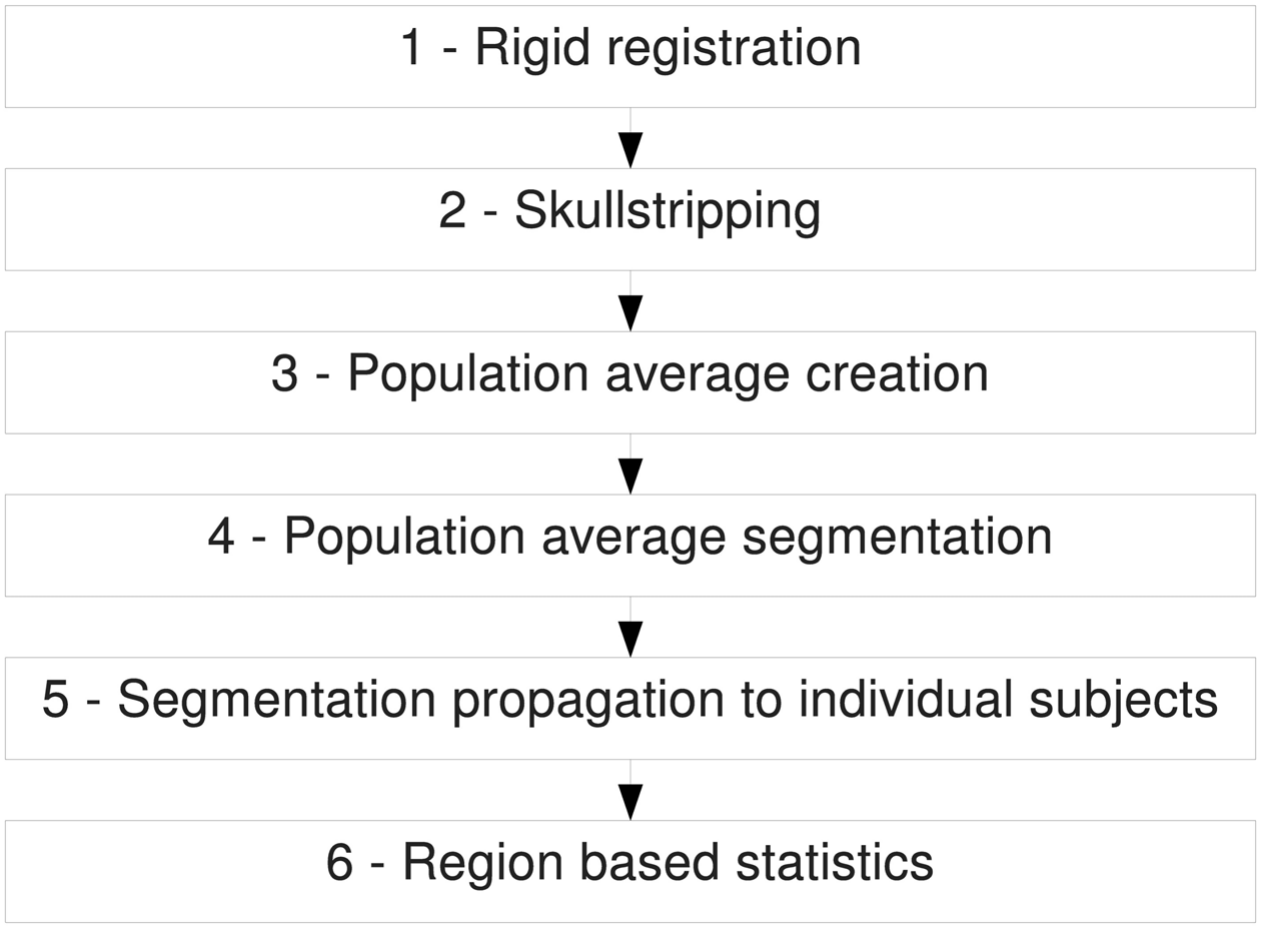
**Overview of the entire pipeline**.

The pipeline is composed of six major steps (Figure 2): Rigid registration, skull-stripping, creation of a population average, parcellation of the population average, parcellation propagation to individual subjects and region based statistics. In some of those steps, existing software developed in other laboratories has been integrated to the pipeline we present here. Those steps are marked with an asterisk (^*^) in the figures and the software is cited in the description of the step. The order of these steps matters since each of them depends on the output of the previous one. For most of the steps, every input image will be processed individually and independently. This allows parallel processing when possible. The images acquired by the scanner are often in DICOM or in raw format. Once they are converted into a format readable by the Insight Toolkit Library^10^ (ITK), on which most tools used in the pipeline are based, one can start processing them using the presented pipeline. Each step is described in detail below.

### Rigid registration (step 1)

**Figure 3:**
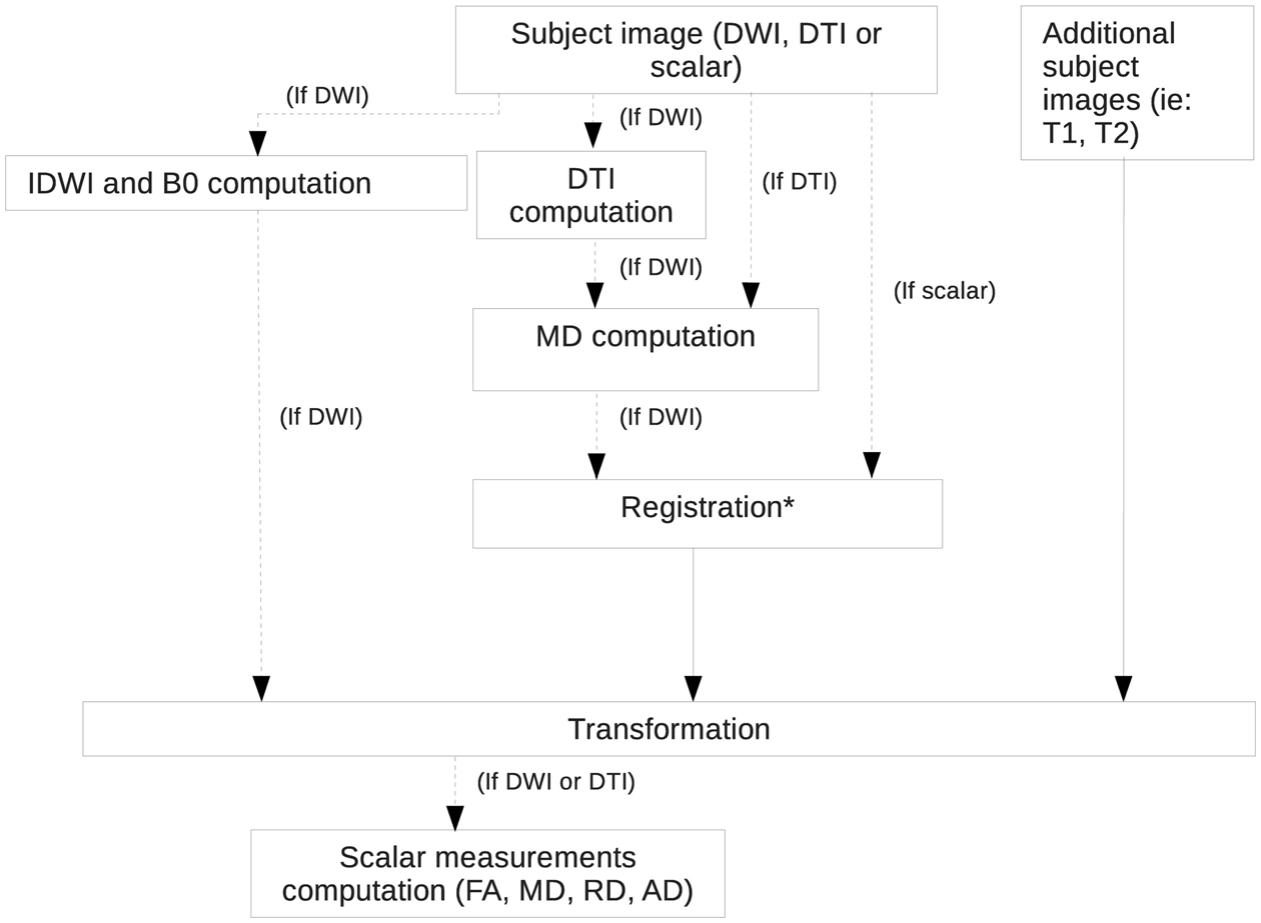
**Rigid registration pipeline**. Registration is performed using RegisterImages, a tool distributed with 3D Slicer.

The first step of the pipeline consists of aligning all the images with a template, such as the one available with the C57 Brookhaven atlas (Ma et al., 2005). Additional details about available templates and atlases are given in Parcellation of Population Average (Step 4). As shown in Figure 3, the necessary inputs to this step are a template image and a subject image, which can be either a DTI, a DWI or an sMRI scan. This step allows the researcher to easily compare the results of the different images in a common coordinate space as well as to perform the following steps. Only a rigid registration, which computes a transformation composed of a translation and a rotation, is performed to keep the size of the brain constant in order to compare brain and region volumes. Performing another type of registration such as an affine registration or other with higher order transformations would result in deforming the original brain, possibly scaling the image and therefore modifying the original volumes of the brain regions.

Before the registration is computed, an intensity bias field correction is performed on the data using N4ITKBiasFieldCorrection (Tustison et al., 2010), a tool distributed with 3D Slicer^11^ (Gering et al., 1999; Pieper et al., 2004, 2006), a multi-platform software for visualization and medical image computing. The registration is performed using RegisterImages (“Registration” step in Figure 3), also part of 3D Slicer package. If the input image is a structural MRI scan (T1, T2, etc), it is simply registered to a given template. If no template is available to the user, one can use a reference image that has been manually oriented correctly. If the input image is a DWI scan, a DTI scan will be estimated from it. The DTI scan will be used to compute the MD image which is then registered to the chosen template. Once the transformation is computed, it is applied to the DTI scan which is then resampled using the log-Euclidean framework (Arsigny et al., 2010). The b = 0 and iDWI images, if present, are also transformed. Finally, all of the derived images (MD, FA, RD, AD) are computed from the registered DTI data. Any given additional images (i.e., T1, T2) are also resampled using the computed transformation. They are expected to be aligned with the input image. Therefore, one has all the input and related images aligned with the template.

### Skull-stripping (step 2)

**Figure 4:**
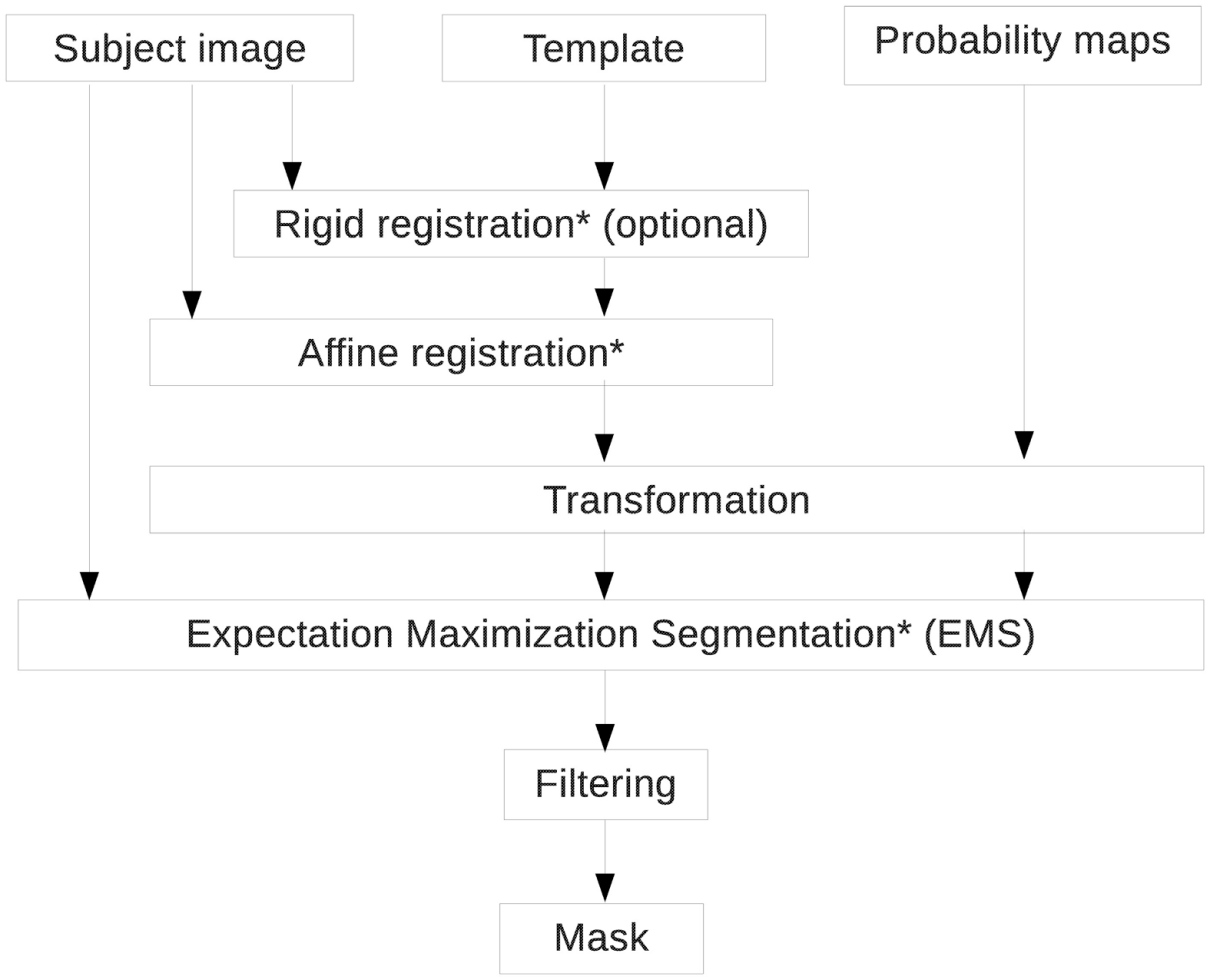
**Skull-stripping pipeline**. Registrations are performed using RegisterImages and Expectation Maximization Segmentation (EMS) is computed with Atlas Based Classification (ABC). Both tools are available in 3D Slicer.

Next, the image is skull-stripped in order to remove the extraneous tissues that are not part of the brain (Figure 4). In the current pipeline, the method presented by Lee et al. (2009) and Oguz et al. (2011) was implemented. To perform this operation, we first perform an affine registration of a template to each image with specific parameters for rodent images. The template image is not provided as part as the pipeline, but several templates (as well as their parcellation map) are publicly available, such as the C57 Brookhaven atlas (Ma et al., 2005), the Mouse BIRN atlas (MacKenzie-Graham et al., 2006) and the Waxholm atlas (Jiang and Johnson, 2011). The direction of the transformation is chosen so that the output mask, obtained at the end of this step, is in the input image space (i.e., aligned with the input image, typically the output of step 1), and not in the template space (i.e., registered with the template). We then transform a probabilistic tissue segmentation atlas associated with the template image, that can be subsequently used for tissue segmentation, into the input image space using the computed transformation. Atlas Based Classification^12^ (Prastawa et al., 2005), also known as ABC, a tool that implements the Expectation-Maximization Segmentation (EMS) algorithm (Pohl et al., 2007), classifies the different tissues of the brain into white matter (WM), gray matter (GM), cerebrospinal fluid (CSF) and the rest of the image, using the transformed probabilistic tissue segmentation maps. GM, WM and CSF are then combined to generate a whole-brain mask. It is then filtered: the largest connected component of the obtained segmentation is selected to remove voxels outside the brain wrongly classified (i.e., false positives) and a binary closing operation is performed to smooth the boundaries and get rid of small regions erroneously classified as non-brain inside the brain (i.e., false negatives). The resulting mask will be used in the following steps of the pipeline. The step necessitates an input image (DWI, DTI or sMRI scan), a template image and corresponding probabilistic tissue segmentation as inputs. The output will be a brain mask.

### Population average creation (step 3)

**Figure 5:**
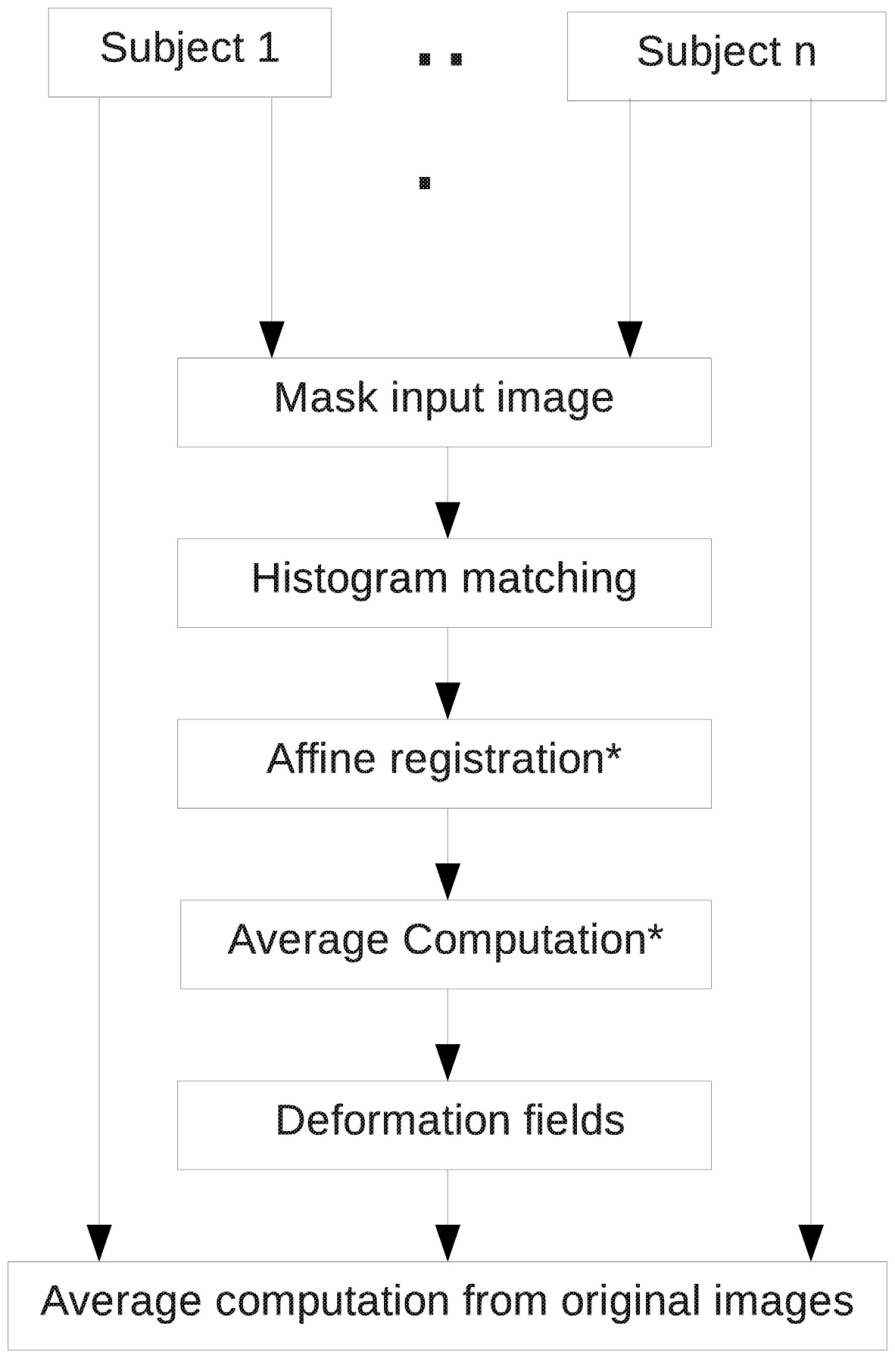
**Population average computation pipeline**. Registration is performed using RegisterImages (distributed with 3D Slicer) and Average Computation is done with AtlasWerks.

Once the images are skull-stripped, the computation of a population average image (Figure 5) is possible. For each case, the mask that was computed at the previous step is applied to the corresponding subject image used for this step. First, all the skull-stripped images are histogram-matched and affinely registered to the first case image. A first average image is computed. However this could create bias based on ordering of images. To limit this effect, we register affinely all the images a second time to the coarse average image just obtained. Once this is completed, we compute an unbiased atlas using a fluid-based registration algorithm performing voxel-by-voxel diffeomorphic mapping (Joshi et al., 2004) using AtlasWerks^13^. This generates an average image as well as deformation fields that transform each image to the average image. To limit smoothing due to multiple interpolations, we recompute the average image from the original images after merging, for each case, the rigid transform obtained in the first step with the deformation field just computed. This way only one interpolation is performed instead of two resulting in a sharper population average image. In the case where the input images are Diffusion Tensor (DT) or Diffusion Weighted (DW) images, an average DT image is computed.

The step additionally creates, for each input image, a deformation field that corresponds to the inverse diffeomorphic registration that was computed to create the average image. This inverse deformation field can be used in step 5 of the pipeline, depending on the selected options.

### Parcellation of population average (step 4)

To avoid having to manually segment the different regions of the brain in the population average that was computed previously (output of Step 3), we use a parcellation associated with an external template. As mentioned in Skull-Stripping (Step 2), several atlases are publicly available. A diffeomorphic registration between the chosen external template and the average image is performed using ANTs (Avants et al., 2008). The obtained transformation is applied to the external parcellation map. This creates the output label map corresponding to the population average parcellation.

### Parcellation propagation to individual subjects (step 5)

The parcellation of each individual is useful to compare differences across subjects or across populations. One already has the transformations (inverse deformation fields obtained at Step 3) to propagate the population average parcellation to all the cases that were used in step 4. If all the cases in the study were used to compute that image, one just has to use those transformations to obtain the parcellation of each scan. Otherwise, the population average has to be registered to each image using a diffeomorphic algorithm. The obtained deformation fields are then used to warp the average image parcellation to the individual cases.

### Region based statistics (step 6)

Once each subject is segmented, computation of whole brain and regional statistics is possible on all the available images, using their brain mask and their individual parcellation respectively. This includes volumes, means and standard deviations of the intensity in the segmented regions or over the whole mask. For diffusion images, this gives the values for the diffusion properties of the image such as FA, MD, RD, and AD.

## Data and analysis

To illustrate our pipeline's functionality, we applied it to a small data set composed of two groups of mouse brain images. All animal treatment protocols were approved by the University of North Carolina at Chapel Hill Institutional Animal Care and Use Committee. The two groups were scanned with different head coils. We then conducted a short statistical analysis to measure the differences between the two sets of scans. The experimental part was used to present the functionalities of the pipeline, and it was not intended to present novel clinically oriented results. This pipeline has been used to process data for other studies with a clinical intent that have been published (O'Leary-Moore et al., 2012).

### Images: acquisition

Two groups of 45 days old mice were scanned on a 9.4 T vertical bore Oxford magnet with shielded coils. For both groups, the image matrix was 512 × 256 × 256 over a 22 × 11 × 11 mm field of view giving 43 × 43 × 43 um isotropic voxels (Jiang and Johnson, 2011). The repetition time was 100 ms. The echo time was 11.828 ms. The bandwidth was 61.5 KHz. The diffusion pulses were two 1.3 ms sinusoid pulses separated by ~6.15 ms (start to start). The scans were acquired with six gradient directions and one b = 0 (gradient amplitude = 1500 mT/m). A total of fifteen mice were imaged. Each individual of the first group (A) was imaged with a solenoid radiofrequency coil that was built in-house, constructed from a single sheet of microwave substrate, twice to increase the signal-to-noise ratio (SNR). Eleven images were acquired. The second group (B) was imaged with a different custom radiofrequency coil which produced scans with higher SNR. Each individual mouse in the second group was therefore scanned only once, with the original imaging protocol. Four images were acquired for the second group.

### Result analysis

We used the developed pipeline to compare the two groups of scans. Each input image was processed using the developed pipeline and we applied a simple *t*-test between the two groups to detect if there was any statistical difference between them (no multiple comparison correction was applied). Statistical significance was defined as *p* < 0.05. Though we understand our sample size is small, we present results in this paper to demonstrate how a *t*-test could be applied to a larger dataset processed with this processing pipeline.

Data distribution (Figures 12, 13) is shown with box and whisker diagrams including the median (bar band), the 25th and 75th percentile (left and right bar respectively) and the minimum and maximum (whiskers).

## Results

**Figure 6:**
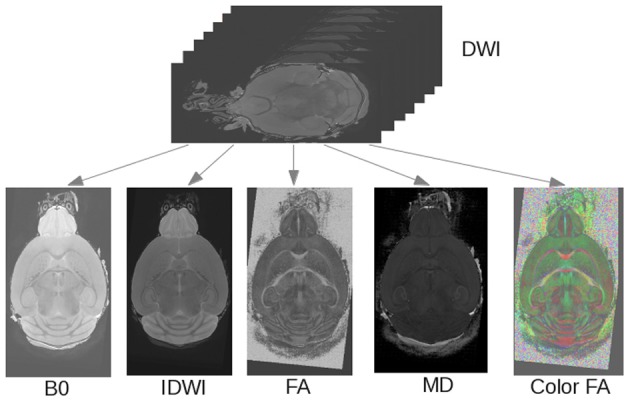
**Rigid registration pipeline**. The DWI scan is registered to a template and derived images (MD, FA, b = 0, iDWI, color-coded FA) are computed.

**Figure 7:**
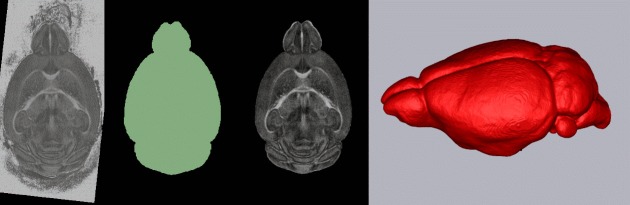
**Skull-stripping pipeline**. From left to right: original FA image, computed mask, original FA image skull-stripped, 3D rendering of computed mask.

**Figure 8:**
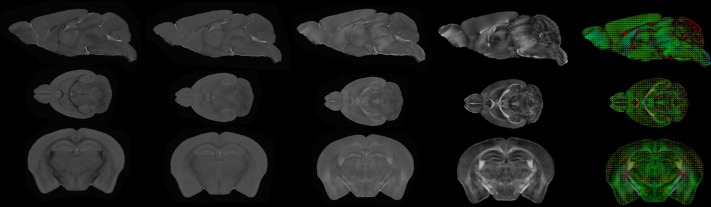
**Population average computation pipeline output**. From top to bottom: sagittal view, axial view, coronal view. From left to right: RD image, MD image, AD image, FA image, color-coded FA with ellipsoids representing the tensors superimposed.

**Figure 9:**
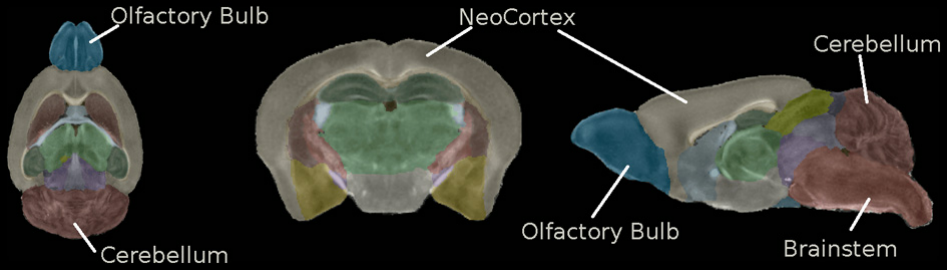
**Regional segmentation of the population average FA image**. From left to right: Axial view, coronal view, sagittal view. The list of the different regions is: Hippocampus, Corpus Callosum and External capsule, Caudate and Putamen, Anterior commissure, Globus Pallidus, Internal capsule, Thalamus, Cerebellum, Superior Colliculi, Ventricle, Hypothalamus, Inferior colliculi, Central Gray, Neocortex, Amygdala, Olfactory Bulb, Brain Stem, Rest of Midbrain, Basal Fore Brain and Septum, Fimbria, Pituitary.

**Figure 10:**
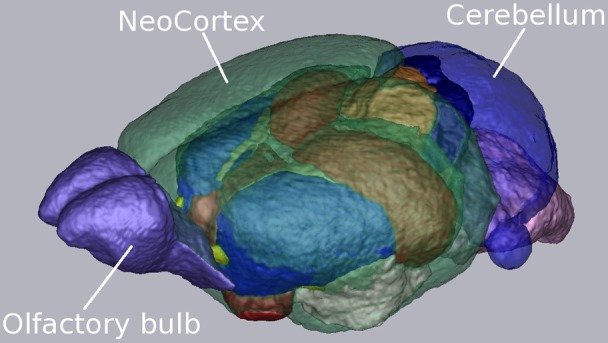
**3D rendering of the population average segmentation**. Cerebellum, neocortex, corpus callosum and external capsule were made partially see-through to allow better visualization of the inside regions.

The visualizations presented in the following section (Figures 6–10) were obtained using 3D Slicer as well as ITK-SNAP^14^ (Yushkevich et al., 2006), a software application used to segment and visualize structures in 3D medical images.

### Rigid registration

The first step of the pipeline rigidly registers each input image to a template (Figure 6). In this analysis, the input image was a DWI scan. A DT image was estimated from it, and its MD was computed to be registered to the given template (in this case, we used the C57 Brookhaven atlas). Once the transformation was computed, it was applied to the DTI data, the iDWI and the b = 0 images that were computed from the DWI scan. The FA, MD, and color-coded Fractional Anisotropy (cFA) were then computed from the transformed DTI data. The cFA is an image in which at each voxel the orientation of the tensor is coded with a color (green: anterior-posterior; blue: superior-inferior; red: left-right) that is modulated by the intensity of the FA.

### Skull-stripping

A mask of the brain is computed using the rigidly registered image. In Figure 7, we present the result of this step for one case. The mask is applied to the FA image and is rendered in 3D.

### Computation of the population average

The next step of the pipeline computes the population average of the input images. Figure 8 presents the scalar images (MD, FA, RD, AD) estimated from the obtained average DTI scan as well as the cFA overlaid with the tensors represented as ellipsoids.

### Segmentation of population average

In the current study, we used the C57 Brookhaven atlas as the external template and atlas. This pipeline step diffeomorphically registered the external template to the population average and applied the obtained transform to the C57 Brookhaven atlas. Results are shown in Figures 9, 10.

### Segmentation propagation to individual subjects

**Figure 11:**
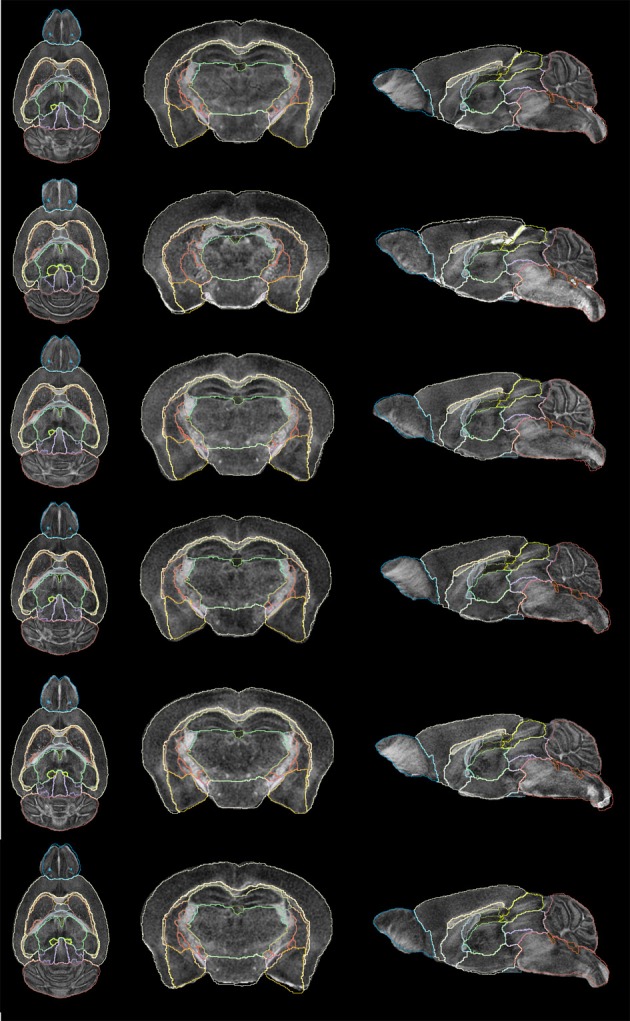
**Subject segmentation**. From left to right: axial view, coronal view and sagittal view of cases 1–6 (bottom–top).

To propagate the segmentation to the individual subjects, the inverse deformation fields were not used in this experiment. The registration between the population average image and each individual were recomputed for another study including cases not used to compute the population average. The new deformation fields were applied to the population average segmentation. Results are shown in Figure 11 for all subjects.

### Statistical analysis of the pipeline output

We applied a *t*-test to compare the two populations that were scanned with different head coils (population A: cases 1–11; population B: cases 12–15). By comparing those two groups of scans we can assess whether there is a significant difference in the images due to the new head coil. The power of this specific statistical analysis is low due to the low number of samples in each group, especially for the *t*-test. This analysis is presented in this paper only to illustrate the possibilities opened by using this pipeline to analyze data.

Table 1 contains the whole brain statistics of the processed images. The volumes of the two groups seem to match well when comparing the whole brain. The AD, MD and RD are statistically different between the two groups (*p* < 0.05). The FA does not present any statistical differences either. This can possibly be explained by the fact that FA is a ratio of the diffusion along the different directions. Even though the absolute values of the measured diffusion amounts are changed, it is plausible that their relative magnitudes captured by FA remain stable.

**Table 1:** **Whole brain volume (mm^3^); average MD (10^−4^ mm^2^/s), FA, RD (10^−4^ mm^2^/s) and AD (10^−4^ mm^2^/s) intensities; *t*-test to compare group A (subjects 1–11) to group B (subjects 12–15)**.

**Subject (group)**	**Volume (in mm**^**3**^**)**	**MD**	**FA**	**RD**	**AD**
1	489.1	4.490	0.3222	3.698	6.074
2	479.8	5.733	0.2767	4.890	7.42
3	490.1	5.249	0.3258	4.344	7.058
4	493.4	5.408	0.2931	4.562	7.100
5	468.2	4.606	0.3193	3.827	6.163
6	469.4	4.696	0.3183	3.897	6.295
7	469.0	5.378	0.2993	4.539	7.056
8	462.0	4.943	0.3049	4.144	6.542
9	500.4	4.449	0.3499	3.605	6.136
10	463.1	4.806	0.3218	3.961	6.496
11	489.7	5.148	0.3189	4.253	6.936
12	481.3	7.985	0.3001	6.692	10.570
13	468.1	6.061	0.3524	4.889	8.406
14	478.0	5.018	0.3486	4.070	6.914
15	489.1	6.107	0.3553	4.968	8.383
*t*-Test	0.9646	0.0071^*^	0.0596	0.0187^*^	0.0017^*^

Statistically significant differences (p < 0.05) are marked with an asterisk (^*^).

**Figure 12:**
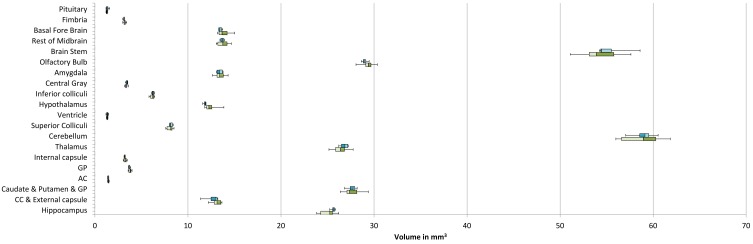
**Distribution of the segmented brain region volumes (mm^3^)**. Neocortex volume is not presented for scale difference reason. Median: bar band; 25th and 75th percentile: left and right bar respectively; minimum and maximum: whiskers.

Among the individual regions of the brains that were segmented using the developed pipeline, no statistical differences in volume between the two groups were found (Table 2). Figure 12 illustrates the volume distribution of the segmented brain regions.

**Table 2:** ***t*-Test of the regional differences between the two groups**.

***t*-Test**	**Volume**	**AD**	**FA**	**RD**	**MD**
Hippocampus	0.161785	0.002171^*^	0.083883	0.014185^*^	0.006480^*^
CC & External capsule	0.176126	0.002440^*^	0.945476	0.035062^*^	0.010841^*^
Caudate & Putamen & GP	0.967358	0.002652^*^	0.012038^*^	0.034600^*^	0.01245^*^
AC	0.58113	0.004561^*^	0.895986	0.015399^*^	0.007892^*^
GP	0.357158	0.004783^*^	0.118830	0.065355	0.023774^*^
Internal capsule	0.543368	0.002063^*^	0.636858	0.044567^*^	0.01155^*^
Thalamus	0.267541	0.002253^*^	0.034974^*^	0.028814^*^	0.010214^*^
Cerebellum	0.807982	0.002723^*^	0.775615	0.016528^*^	0.00778^*^
Superior colliculi	0.449734	0.002356^*^	0.550493	0.005228^*^	0.00363^*^
Ventricle	0.744692	0.060377	0.097752	0.323671	0.187511
Hypothalamus	0.078088	0.001211^*^	0.039063^*^	0.011286^*^	0.004133^*^
Inferior colliculi	0.494573	0.004877^*^	0.482485	0.007751^*^	0.006061^*^
Central gray	0.277711	0.002421^*^	0.151546	0.043269^*^	0.014244^*^
Neocortex	0.485982	0.002684^*^	0.105912	0.016225^*^	0.007889^*^
Amygdala	0.858912	0.002583^*^	0.065014	0.010802^*^	0.005608^*^
Olfactory bulb	0.355956	0.000748^*^	0.148953	0.012075^*^	0.003904^*^
Brain stem	0.371267	0.005558^*^	0.043845^*^	0.110809	0.032877^*^
Rest of midbrain	0.925355	0.001040^*^	0.027451^*^	0.024848^*^	0.006166^*^
Basal fore brain	0.278086	0.002233^*^	0.099579	0.017967^*^	0.007121^*^
Fimbria	0.221511	0.000912^*^	0.832303	0.039053^*^	0.007191^*^
Pituitary	0.291476	4.62E−05^*^	0.237743	0.007372^*^	0.000587^*^

Statistically significant differences (p < 0.05) are marked with an asterisk (^*^).

**Figure 13:**
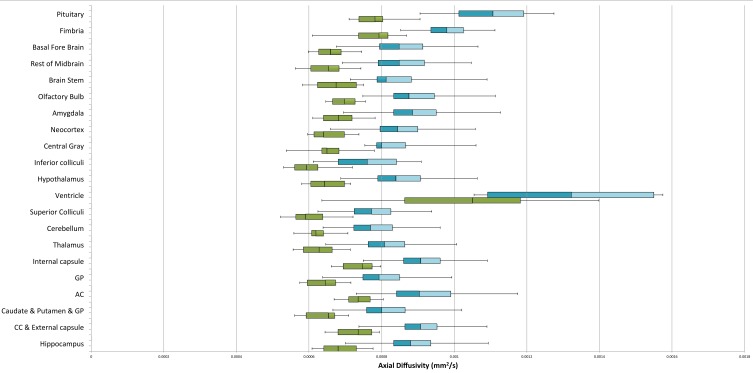
**Axial diffusivity (mm^2^/s) of the segmented brain regions**. Median: bar band; 25th and 75th percentile: left and right bar respectively; minimum and maximum: whiskers.

For all segmented brain regions but the ventricles, the significant statistical difference in the AD between the two groups (Table 1) over the whole brain is also present in individual regions (Table 2). Figure 13 shows the AD value distribution of the segmented brain regions. FA does only present statistical differences for certain regions such as the thalamus, the hypothalamus and a few others (Table 2). As explained above, this might be due to the fact that FA is a ratio of the diffusion along the different directions. Finally, RD and MD also present significant differences for most segmented regions (Table 2).

The results presented here are meant to serve only as a proof of concept to illustrate our Midas pipeline. A more thorough statistical analysis, including a larger sample size and correction for multiple comparison tests, is necessary before drawing any conclusions from this data set.

## Discussion

The pipeline presented herein allowed automated processing and comparison of two groups of mouse images scanned with different head coils. Visual inspection by a human expert confirmed that the results after each step of the pipeline were satisfactory. We were able to align the images, skull-strip them, compute a population average, segment the population average, propagate the segmentation to every individual and compute statistics over each individual image (MD, FA, AD, RD). The computation of these statistics was completed for the whole brain, using the brain mask, as well as for each segmented region, using the warped segmentation map from each case.

It is possible that in some circumstances, one want to skip some steps of the processing pipeline. For example, if one is only interested in skull-stripping the images (e.g., only measure the brain volume), registration of the images rigidly to an external template may not be necessary (step 1). Indeed, in step 2, the template is registered, rigidly and affinely, to the subject to transform the probability maps from the template space to the subject space, to apply the EM algorithm. However, there are three problems in skipping that step: (1) if the input images are DTI or DWI scans, one will still need to compute scalar images derived from those for the skull-stripping step to be achieved; (2) the skull-stripping results are usually more accurate after rigid registration; (3) the result of Step 3 (creation of a population average) is sharper if the rigid registration has been computed due to a better alignment.

The computation and segmentation of a population average are also optional. One may want to directly warp the external template segmentation to each individual image directly. This may be necessary in situations where the appearance of the individuals is too diverse for a stable population average to be computed. Additionally, computing only a single diffeomorphic registration directly to the external atlas may give, in some cases, a better result than having two warping steps (external template to population average followed by population average to individual). However, adding the population average creation and segmentation steps gives less biased results. If comparing two populations, one being close to the external atlas, and one being very different, the results obtained using direct registration of the external template to each individual will be biased. However, if one first computes an average using both populations and then registers this newly computed average to each subject, the result will be less biased, the average image including subjects from both populations.

Additionally, the population average image has a better SNR and therefore the registration of the external template to this image is usually more robust than the one to the individual images.

After each step, if one is not satisfied with the results obtained by the automatic processing, one can manually correct the images. For example, if the skull-stripping (step 2) is not accurate enough, one can edit masks that are not precise enough and use those corrected images in the rest of the pipeline. This also holds for the rigid registration (step 1) and the population average segmentation (step 4) steps as well. If one is not satisfied with the rigid registration results, one can directly provide a manual transformation to the pipeline and skip the computation of the automatic transformation. In such a case, the registration step will still compute the transformed image as well as the derived images if any (Fractional Anisoptropy, MD, etc.). This is useful when processing DWI and DTI.

It is possible to conduct additional processing of the data after the pipeline has been run. For example, a voxel-by-voxel analysis (Li et al., 2010) like those reported in previous studies (Tyszka et al., 2006; Van Camp et al., 2009) can be performed on the individual subjects transformed into the population average space using the deformation field transforms. Fiber tract-based analysis can also be used to detect white matter abnormalities (Sizonenko et al., 2007; Zhang et al., 2007) and to analyze DTI scans (Huang et al., 2004; Asanuma et al., 2008). These analyses can be performed in both the individual images, or in the population average space (Goodlett et al., 2009) which has a better SNR and propagated to individual images using the deformation field transforms computed with the pipeline. In addition to fiber tract analyses, assessment of cortical thickness (Tamnes et al., 2010; Lee et al., 2011) can be done in this type of analysis: the cortical thickness in the population average is computed and propagated the result to each subject again using the deformation field transforms obtained with the pipeline. Deformation field analysis (Walhovd et al., 2010), also called Tensor Morphometry, which allows detection of an increase or a decrease in the volume of a region by analyzing the amount of deformation, can also be performed by analyzing the deformation field computed by the pipeline.

Finally, the Midas platform allows easy collaboration by sharing data and tools. It is not necessary for data to be transferred back and forth between multiple laboratories and/or institutions. Any modified or newly computed data is directly available to each party sharing the platform and the data. Similarly, every institution sharing one Midas platform can share tools enabled by the plugin system without having the usual problems arising such as verifying that the correct versions are used and verifying that those tools are available for the different environments used in all collaborating institutions. An important side effect of this centralization is that input data and results can be better managed to ensure proper data provenance and experimental reproducibility. The web-based interface allows a user-friendly experience and the server-side processing reduces the need for powerful computers at each user's location. This is crucial for allowing biomedical scientists with limited image processing experience to use the DTI technology in their research.

## Conclusion

We presented a pipeline that is freely available^15^ as a plugin for the Midas platform and that can be used to process rodent MR images. We showed preliminary results obtained with such analysis on a sample data set and demonstrated how useful and easy it is to use this pipeline to process images from beginning to end. The current scripts can also be easily adapted for future studies. Additional analysis such as voxel-by-voxel analysis, cortical thickness analysis, deformation field analysis or tractography can also be performed using the results of the pipeline or by adding a step to the current pipeline. Those additional steps will be included in the pipeline in later versions.

### Conflict of interest statement

The authors declare that the research was conducted in the absence of any commercial or financial relationships that could be construed as a potential conflict of interest.

## Footnotes

^1^FSL is a comprehensive library of analysis tools for FRMI, MRI and DTI brain imaging data: http://www.fmrib.ox.ca.uk/fsl/

^2^The LONI Pipeline is a free workflow application primarily aimed at computational scientists: http://pipeline.loni.ucla.edu/

^3^BrainSuite is a suite of image analysis tools designed to process magnetic resonance images (MRI) of the human head: http://www.loni.ucla.edu/Software/BrainSuite

^4^FreeSurfer is a set of automated tools for reconstruction of the brain's cortical surface from structural MRI data, and overlay of functional MRI onto the reconstructed surface: http://surfer/nmr.mgh.harvard.edu/

^5^AtlasWerks is a set of high-performance tools for diffeomorphic 3D image registration and atlas building. Scientific Computing and Imaging Institute (SCI).

^6^The ANTs package is designed to enable researchers with advanced tools for brain and image mapping: http://www.picsl.upenn.edu/ANTs/.

^7^Mni_autoreg are a set of tools to automatically linearly and nonlinearly register two volumes together: https://github.com/BIC-MNI/mni_autoreg

^8^http://batchmake.org

^9^The Midas Platform is an open-source toolki that enables the rapid creation of tailored, web-enabled data storage: http://www.midasplatform.org

^10^ITK is a widely used C++ library designed for image processing purposes: http://itk.org

^11^http://www.slicer.org

^12^http://www.nitrc.org/project/abc/

^13^http://www.sci.utah.edu/sofware.html

^14^http://www.itksnap.org/

^15^URL to download the rodent pipeline plugin for the Midas platform: https://github.com/midasplatform/rodent
